# Draft Genome Sequence of *Streptomyces iranensis*

**DOI:** 10.1128/genomeA.00616-14

**Published:** 2014-07-17

**Authors:** Fabian Horn, Volker Schroeckh, Tina Netzker, Reinhard Guthke, Axel A. Brakhage, Jörg Linde

**Affiliations:** a Bioinformatics/Systems Biology, Leibniz Institute for Natural Product Research and Infection Biology, Hans Knoell Institute (HKI), Jena, Germany; b Molecular and Applied Microbiology, HKI, Jena, Germany; c Institute for Microbiology, Friedrich Schiller University, Jena, Germany

## Abstract

*Streptomyces iranensis* HM 35 has been shown to exhibit 72.7% DNA-DNA similarity to the important drug rapamycin (sirolimus)-producing *Streptomyces rapamycinicus* NRRL5491. Here, we report the genome sequence of HM 35, which represents a partially overlapping repertoire of secondary metabolite gene clusters with *S. rapamycinicus*, including the gene cluster for rapamycin biosynthesis.

## GENOME ANNOUNCEMENT

*Streptomyces iranensis* HM 35 is a novel member of the *Streptomyces violaceusniger-S. hygroscopicus* group (1). Based on 16S rRNA gene sequencing, HM 35 is phylogenetically closely related to *Streptomyces rapamycinicus* NRRL 5491, followed by *S. violaceusniger* DSM 40563, *Streptomyces yogyakartensis* DSM 41766, and *Streptomyces javensis* DSM 41764 (1). Thus, this species is of special interest for the exploration of its genomic and chemical capacity, which may include interesting bioactive compounds, such as the antifungal and immunosuppressant drug rapamycin (2), but it also allows for the discovery of novel secondary metabolites and a study of their regulation (3, 4).

Genomic DNA from *S. iranensis* HM 35 (NCBI taxonomy ID, 576784; sample ID, DSM41954) was obtained from a sample cultured in tryptone soya broth (TSB) (5). DNA library preparation (paired-end 2 × 100 bp) and sequencing on Illumina HiSeq 2000 were performed at LGC Genomics (Berlin). The raw reads were adapter clipped (6), quality trimmed, and error corrected (7). The initial contigs were generated using Velvet (8). The contigs were shredded into overlapping 350-bp sequences, generating a coverage of 17×, and assembled using Newbler 2.6 (454 Life Sciences). Gaps in the resulting sequences were filled using the Beijing Genomics Institute (BGI) GapCloser software (9).

For *ab initio* gene prediction, GeneMark-ES (10) was applied. Functional annotation was performed using Blast2GO (11) and InterProScan (12). Gene descriptions were obtained by blasting the predicted protein sequences against those of *Streptomyces bingchenggensis* BCW1 (Genbank accession no. CP002047) and *S. violaceusniger* Tu4113 (Genbank accession no. CP002994). Matches with the lowest e-value below 10^-5^, 70% sequence identity, and a subject hit length of 70% were considered highly similar. Secondary metabolite gene clusters were predicted using antiSMASH (13).

DNA sequencing resulted in 17,692,354 raw reads, of which 16,014,549 reads passed the quality filter (estimated genome coverage, 122.7-fold) and were used for sequence assembly. The resulting assembly consists of 7 scaffolds and 12.1 Mbp (longest scaffold, 12.0 Mbp). The G+C content of the assembly is 70.9%. The final structural gene prediction resulted in 9,967 gene models. We assigned functional names to 7,398 transcripts, GO categories to 4,443 transcripts, and protein domains to 8,359 translated transcripts. A total of 2,120 proteins were predicted to contain transmembrane domains.

AntiSMASH predicted 171 enzymatic genes that may be involved in the synthesis of secondary metabolites. Based on these functional annotations, as well as on a polyketide synthase (PKS) and nonribosomal protein synthesis (NRPS) domain search, the existences of 11 PKS, 5 NRPS, and 3 NRPS-PKS (in total 19) belonging to different secondary metabolite gene clusters were predicted. Noteworthy is the high similarity of the genes SIRAN8002 to SIRAN8065 with the previously described rapamycin gene cluster (14). Indeed, the formation of rapamycin was verified by liquid chromatography mass spectrometry (LC-MS) analysis (data not shown).

### Nucleotide sequence accession numbers.

This whole-genome shotgun project has been deposited in DDBJ/ENA/GenBank under the accession no. LK022848 to LK022854. The version described in this paper is the first version. Genome data and additional information are also available at the HKI Genome Resource (http://www.genome-resource.de/).
